# Unemployment Rate, Smoking in China: Are They Related?

**DOI:** 10.3390/ijerph13010113

**Published:** 2016-01-08

**Authors:** Qing Wang, Jay J. Shen, Chris Cochran

**Affiliations:** 1 School of Business, Dalian University of Technology, Panjin 124221, China; 2 School of Community Health Sciences, University of Nevada Las Vegas, Las Vegas, NV 89103, USA; jay.shen@unlv.edu; 3 Department of Health Care Administration, University of Nevada Las Vegas, Las Vegas, NV 89103, USA; chris.cochran@unlv.edu

**Keywords:** China, current smoker, number of cigarettes smoked daily, unemployment rate

## Abstract

*Background*: Studies on the relationship between unemployment rate and smoking have yielded mixed results. The issue in China has not been studied. This study aims to examine the influence of unemployment rate on smoking in China. *Methods*: Logit model and two-stage least squares (2SLS) estimation were used to estimate the effects. Estimations were done for 4585 individual over 45 using data from China Health and Retirement Longitudinal Study conducted in Zhejiang and Gansu provinces in 2008 and 2012. *Results*: A percent increase in the unemployment rate resulted in the increase in the likelihood of smoking by a combined 9.1 percent for those who smoked including a 2.9% increase for those who smoked 1–10 cigarettes per day; a 2.8% increase for those who smoked 11–20 cigarettes per day; and a 3.4% increase for those who smoked 20 cigarettes or more per day. The effects were stronger for those who were employed. Non-drinkers were more likely to engage in smoking with increased unemployment rate. 2SLS estimation revealed the same association. *Conclusions*: The unemployment rate was positively associated with smoking behavior. Smoking control and intervention strategies should focus on both the individual′s characteristics and the physical environment in which unemployment rate tend to rise.

## 1. Introduction

About 20 percent of the world′s adult population smoke cigarettes [1]. Tobacco caused 100 million deaths over the course of the 20th century [2]. China is the largest producer and consumer of cigarettes [3]. In 2013, China consumed more than 30 percent of the world′s cigarettes and smoking accounts for 1.36 million deaths annually there [4]. As a leading risk factor for global disease burden, smoking consumption is of particular interest [5].

The area-level unemployment rate reflects the macro-economic context/environment, where an individual resides, that may influence her or his smoking behavior [6]. A number of studies, based on data from developed countries, have examined the relationship between area unemployment rate and smoking behavior. The exact nature of the relationship between area unemployment rate and smoking behavior remains unclear due to the mixed results observed in the literature review [6]. Some studies reported that the area-level unemployment rate was associated with an increase in smoking [7,8,9,10], whereas some were null or decreased tobacco consumption [6,11,12,13,14,15,16]. Charles and DeCicca found an increase in smoking among those who are least likely to be employed; however, smoking declined for those in the highest employment decile in U.S. [17]. The spillover effects of high unemployment rates are less well understood [18].

This study, examining the impacts of unemployment rate on smoking in China contributed to the literature in three aspects. First, this study contributed to a better understanding of the relationship in an under-studied Chinese population. No previous studies have focused on the impacts of macroeconomic environments in China [19]. The relationship between smoking and unemployment rate may depend on whether use is examined in developing or developed countries. For example, in developed countries, smokers might consume more in a downturn. This positive relationship found for smoking does not hold for developing countries where disposable income is low [20]. As a developing country, the Chinese macroeconomic policies and environments are drastically different from those of the U. S. and European countries where similar comparisons and implications may not appropriately be made between China and those countries. Second, we attempted to examine the effects of the area-based unemployment rate on smoking of individuals who were either employed or unemployed. Contextual effects of unemployment may exhibit different signs on alternative social groups. Previous studies have calculated an “average” effect of unemployment on smoking behaviors, research is lacking on the effects of unemployment rate on smoking of workforce and non-workforce populations, respectively [18]. In addition, considering cigarette and alcohol are related in consumption, comparative analysis on smoking influence of unemployment rate between current drinkers and non-drinkers was carried out [21,22]. The cross-relation between smoking and alcohol consumption was another point that the previous literature on smoking effects of unemployment rate failed to highlight. Findings showed that the unemployment rate was positively and significantly associated with smoking in China, and the effects were stronger for those who were employment than the entire sample. Drinkers were less likely to smoke when unemployment rate was on the rise.

## 2. Conceptual Framework

Economic factors such as unemployment can lead individuals to change their behaviors without much attention to the potential effects on their personal health [23]. The canonical economic model of health-related decisions derived by Grossman was applied to examine the association between the unemployment rate and smoking [24]. Individuals would consider and make trade-offs between current benefits obtained from risky and unhealthy behaviors and future costs of such behaviors including potentially undesirable consequences related to their health. The decision to smoke can be seen as divestment in the stock of health capital in return for a short-run increase in welfare [25].

Mounting evidence has shown substantial costs being associated with smoking in the long-run. Smokers have higher risks of developing debilitating and often serious illnesses such as cancer, cardiovascular and circulatory diseases, and chronic respiratory diseases. The chemicals in tobacco damage the lungs, blood vessels, and other tissues and keep them inflamed; disrupting the way body heals itself [26]. In short, smoking decreases the individual′s health capital and productivity in the long-run.

However, smokers may experience short-term benefits from smoking, such as relieves in anxiety or appetite suppression [27,28], both of which may help people to ease stresses resulting from possible budget constraints given an environment where the unemployment rate is increasing. For Chinese people, job insecurity due to increased unemployment usually results in stress, anxiety, and psychological hardship [29], one outlet for coping with this stress is to take up or increase smoking [30,31,32]. Besides, smokers may attribute the smoking behavior to unemployment rate, which make smokers be easy to accept behavioral consequences resulting from smoking [33].

Unemployment often provokes a shift from a preference towards the future to a preference leaning towards the present time [34]. Many studies have found that unemployment leads to an erosion of biographical plans, an abandonment of the future and a loss of hope [35,36]. A short-term perspective not only increases the likelihood of seeking short-term rewards that may result in unhealthy behavior such as smoking, but also leads to underestimating the long-term consequences of the unhealthy behavior (e.g., health risks associated with these behaviors).

On the other hand, budget constraints due to increased unemployment may also have opposite effects on smoking [37]. Because unemployment leaves individuals with less discretionary income, individuals may simply decide to abstain or reduce tobacco consumption in order to save money [38]. For example, in China between 1990 and 2005, urban poor families used 7.7% of their reported expenditures on smoking and the rate among rural family was 11.2% [39]. Given that smoking represents a significant share of an individual′s disposable income, higher rates of unemployment may force individuals to reduce or quit smoking.

In addition, the unemployment rate may influence smoking not only on those who have lost their jobs but also on those who remain employed [6]. High unemployment induces a negative externality on the employed. If workers attach their future prospects to their local unemployment rate, anxiety that one might be the next on the list of fired persons will grow with rising unemployment rates.

## 3. Materials and Methods

### 3.1. Data

This study used data from the 2008 and 2012 China Health and Retirement Longitudinal Study (CHARLS) of Zhejiang and Jiangsu provinces. The sample included those who were 45 years old or older at the time of data collection in 2008. There were 2685 individuals in 1570 households in the 2008 sample representing both rural and urban residents. The response rate was 83%. The second survey following the same respondents was conducted in 2012. Newly and randomly sampled respondents added at the 2012 waves of the panel. Observations with missing values were excluded and the final sample size was 4585 individuals surveyed in 2008 and 2012. Biomedical ethics committee of Peking University approved this retrospective study. Written consent was obtained; respondent records/information was anonymized and de-identified prior to analysis.

The CHARLS adopted a multi-stage, stratified Probability-Proportionate-to-Size (PPS) Sampling. Counties were chosen by PPS stratified based on urban or rural regions. Within each county, CHARLS randomly selected three villages based on PPS. Within each village or community, CHARLS then randomly selected 25–36 dwelling units from a complete list of dwelling units generated from a map, determining the number of households in each chosen dwelling unit. Next, CHARLS randomly selected one age-eligible household and then determined the number of age-eligible members within a household and randomly selected one member for the survey. Based on this sampling procedure, about 25–36 households in each village and one or two individuals in each household were interviewed depending on marital status in the household (*i.e.*, one for the single and two for the couple in a household).

The CHARLS questionnaire included the following modules: demographics, family structure/transfer, health status and functioning, biomarkers, health care and insurance, work, retirement and pension, income and consumption, assets (individual and household), and community-level information. The CHARLS is similar to the Health and Retirement Study (HRS) in the United States, the English Longitudinal Study of Aging (ELSA) in the United Kingdom, and the Survey of Health, Aging and Retirement in Europe (SHARE) (for details, see http://charls.ccer.edu.cn/zh-CN).

### 3.2. Measures

#### 3.2.1. Smoking Behavior

Two dependent variables were examined. The first, smoking status, was classified as a current smoker or a non-smoker. The second, “Number of Cigarettes Smoked Daily”, was based on the responses of CHARLS question “How many cigarettes do you smoke each day now?” “Number of Cigarettes Smoked Daily” was recoded into an ordinal variable from 1 to 4 representing the level of smoking (1 = non-smoker at present; 2 = smoked cigarettes 1–10 per day; 3 = 11–20 cigarettes per day; 4 = more than 20 cigarettes per day). Such a measure of smoking could capture the intensity of smoking dependence among respondents, which may not be obtained in the present widely used binary indicator of being a current smoker.

#### 3.2.2. City-Level Registered Unemployment Rate

The independent variable was the annual city-level registered unemployment rate. It was calculated by taking the average of the monthly city level unemployment rates of 2008 and 2012, respectively. The monthly unemployment rate information was obtained from National economy and society developed statistical bulletin 2008, 2012, which is publicly available. As National Bureau of Statistics of People′s Republic of China website presents, the registered unemployment rate refers to the rate between the registered unemployed persons and total labor force. Unemployed individuals, who have not registered as either employed or unemployed, are not regarded as unemployed persons. Therefore, individuals, who were not currently working or were actively looking for work but did not go to the government employment service agencies for registration, were excluded. The registered unemployment rate is lower than the investigated unemployment rate, which is the proportion of all unemployed persons and labor force, and used widely in developed countries. However, in 2008, the registered unemployment rate is the only measure of unemployment rate reported in China.

#### 3.2.3. Individual Socio-Economic Status and Demographic Variables

Several control variables were included when we run the health-related economic decision model, derived by Grossman [24]. Demographic variables included age, gender (reference group: female) and marital status (married was the reference). Education, job status, and income are the most commonly used measures of social economic status in health and smoking behavioral research [40,41]. Educational attainment, in our study, was defined at three levels: primary school or below, junior high school, and senior high school or above. Two dummy variables were created for educational attainment, with primary school or below serving as the reference group. Job status was either unemployed or employed. It is noted that the employed category included those who were employed or retired. Household income divided by the number of household members was controlled. Another dummy variable, “living in urban area”, indicated whether or not an individual lived in an urban area. Respondents were asked if they drank beer or any other alcoholic beverage during the previous 12 months. Those who had drunk in the past 12 months were identified as “current drinker” and asked further questions about: frequency of drinking. Drinking more than 2–3 times a week was considered “drinking frequently” (current drinker was not included in the regression to avoid collinearity). Since tobacco price has been used as a principal tool to discourage smoking, log of cigarette price was also included [16]. Measure of cigarette price was available from CHARLS question “How much does/did it cost per pack = 20 cigarettes?”. We obtained the average price per pack of cigarettes in each city at the time of the survey administration by calculating the means of individual data with cross-section individual weight adjust. All cigarette prices were adjusted for inflation and represent 2008 Yuan.

### 3.3. Analytical Techniques

Since smoking behavior was measured by ordered outcomes, Logit and Ordered Logit models were applied to analyze the relation between area-level unemployment rate and smoking behavior, controlling for the individual socio-economic status, health behavior and demographic variables. The study estimated the following empirical specification:

(1) $$S_{ijt}=\beta_{0}+X_{ijt}\beta_{1}+U_{jt}\beta_{2}+v_{j}+u_{t}+\varepsilon_{ijt}$$

*S_ijt_* represents smoking behavior for individual *i* living in province *j* in year *t*. *X_ijt_* represents a vector of observed individual characteristics, such as age, marital status, education, income, *etc.* *U_jt_* is the average unemployment rate of city in year. *v_j_*, *u_t_* represent city and year specific fixed effect, respectively, and ε*_ijt_* is the error term.

We also took into account the potential clustering factor at the level of city by using Huber–White corrected standard errors.

Unemployment rate was potentially endogenous due to omitted variables and/or simultaneity [42]. We were unable to directly measure factors such as attribution bias, emotion towards unemployment rate, and early experiences, which were unavailable in our database, leading to omitted variables problems [33,43]. In addition, smoking behavior may also affect unemployment, leading to a simultaneity issue [44,45]. In order to address the endogeneity problem, this study employs the two-stage least squares (2SLS) estimation approach with a valid instrument variable. The instrument used in this study was log of inflation-adjusted area-level unemployed insurance gold. Area-level unemployed insurance gold ranged from 300 to 1048 in our study; the mean value is 610 Yuan. This instrumental variable should be correlated with unemployment rate, but should not directly affect smoking behavior and only work indirectly through its effects on unemployment rate. Unemployed insurance gold is the significant step that ensures unemployed personnel to live basically, also be the situation that organization of unemployed insurance agency understands unemployed personnel in time, promote its to come true as soon as possible again the significant step of obtain employment [46]. As expected, unemployed insurance gold was negatively correlated with area-level unemployment rate (the results are available at request). The instrumental variable was significant at the 1% level by an *F* test. We performed several other tests to verify the validity of these instruments, including: test of excluded instruments, under-identification test, weak-identification test, and weak-instrument-robust test. The *p* values for these tests are statistically significant at the 1% level, indicating that our instruments are valid. All analyses were performed using the STATA version 12.0.

## 4. Results

The average city-level unemployment rate was 3.19% with 3.4% in the wave of 2008 and 2.95% in the wave of 2012. Variations in the unemployment rate existed across cities, with the lowest being 2.50% in Wenzhou of Zhejiang province and the highest being 3.91% in Linxia of Gansu province in 2008; with the lowest being 1.63% in Hangzhou of Zhejiang, the highest being 3. 68% in Dingxi of Gansu in 2012.

Table 1 shows descriptive statistics for the variables. The average age of current smokers was 61 years. Thirty-one percent of the respondents identified themselves as smokers: 6 percent smoked fewer than 10 cigarettes per day, 8 percent smoked more than 10 but fewer than 20 cigarettes, and 17 percent smoked more than 20 cigarettes daily. The distribution of smokers in the CHARLS data was very similar to the distribution in China report on health hazards of smoking [47]. Table 1 also shows socioeconomic and lifestyle characteristics of individuals who were employed. After excluding the unemployed, the sample size was down to 3908, among them 35 percent identified themselves as smokers: 7 percent who smoked fewer than 10 cigarettes a day, 9 percent who smoked more than 10 but fewer than 20 cigarettes, and 19 percent who smoked more than 20 cigarettes a day. The smoking prevalence was higher among those who were employed than that of the entire sample.

Table 2 shows the multivariable regression results of the relationship between the area unemployment rate and individual smoking behavior after controlling for socio-economic status, alcohol consumption and other demographic characteristics. The positive association supported the hypothesis that the unemployment rate had positive impacts on smoking behavior in China.

Table 3 presents the marginal effects of unemployment rate on smoking behavior in China. In the Logit model, the unemployment rate increased the likelihood of smoking by 10 percent. In the Ordered Logit model, where the dependent variable was an ordinal variable representing the decision to smoke or not and how much, the unemployment rate increased the likelihood of smoking by 9.1 percent. The unemployment rate increased the likelihood of smoking 1–10 cigarettes daily by 2.9 percent; the likelihood of smoking 10–20 cigarettes daily by 2.8 percent; and the likelihood of smoking more than 20 cigarettes by 3.4 percent.

**Table 1:** Individual socioeconomic and life style characteristics.

Demographics	All Sample		Employed People	
	Mean or N	Std. Dev. or %	Mean or N	Std. Dev. or %
Age	61.1	10.2	61.8	10.6
Male	2247	49	2071	53
Unmarried	779	17	704	18
Educational attainment				
Primary school and below	3622	79	1602	41
Middle school	550	12	1251	32
High school and above	413	9	1055	27
Economic status				
Individual income	12,333.93	16,285	12,490	15,513
Employed	3908	85	3908	100
Urban	963	21	782	20
Tobacco price	5.54	2.33	5.59	2.33
Life style				
Current drinker	1559	34	1211	31
Drink frequently	734	16	625	16
Smoking status	1421	31	1368	35
1-10 a day	275	6	273	7
10-20 a day	367	8	352	9
More than 20 a day	779	17	743	19
Obs	4585		3908	

**Table 2:** Effects of unemployment rate on smoking behavior.

Variables	Smoking		Number of Cigarettes Smoked Daily	
	Cof. Std. Error		Cof. Std. Error	
Unemployment rate	0.66 ***	0.23	0.69 ***	0.22
Tobacco price	−0.30 **	0.14	−0.61 ***	0.18
Age	−0.58 ***	0.31	−1.19 ***	0.34
Male	1.61 ***	0.35	4.48 ***	0.19
Unmarried	0.08	0.11	0.16	0.11
Educational attainment				
Middle school	−0.03	0.10	−0.28 **	0.12
High school and above	−0.13	0.13	−0.32 **	0.14
Household income	1.13 × 10^−7^	1.86 × 10^−7^	1.47 × 10^−7^	9.90 × 10^−8^
Employed	0.40 **	0.16	0.40 ***	0.15
Living in urban area	−0.15 *	0.08	−0.08	0.14
Drink frequently	0.55 ***	0.12	0.37 ***	0.12
Obs	4585		4585	
Prob > chi2	*p* < 0.01		*p* < 0.01	

* *p* < 0.1, ** *p* < 0.05, *** *p* < 0.01; Huber-White corrected standard errors.

**Table 3:** Marginal effects of unemployment rate on smoking behavior.

Variables	Marginal Effect
Smoking (*n* = 4585)	0.10 ***
Number of Cigarettes Smoked Daily (*n* = 4585)	
Non-smoker	−0.091 ***
1–10 a day	0.029 ***
10–20 a day	0.028 ***
More than 20 a day	0.034 ***

*** *p* < 0.01; Huber-White corrected standard errors.

Table 4 shows effects of unemployment rate on smoking behavior of employed individuals. The area unemployment rate was positively associated with smoking behavior, consistent with results obtained from the entire sample. Table 5 presents the marginal effects of the unemployment rate on smoking behavior among employed individuals. In the Logit model, the unemployment rate increased the likelihood of smoking by 13.6 percent. In the ordered logic model, one percent increase in the unemployment rate resulted in the increase in the likelihood of smoking about 10.8 percent comprised of compounded increases in those smoking 1–10 cigarettes per day by 3.3 percent; those smoking 11–20 cigarettes per day by 3.3 percent; and those smoking 20 cigarettes or more by 4.2 percent.

**Table 4:** Effects of unemployment rate on smoking behavior of employed individuals.

Variables	Smoking		Number of Cigarettes Smoked Daily	
	Cof. Std. Error		Cof. Std. Error	
Unemployment rate	0.74 ***	0.22	0.72 ***	0.23
Tobacco price	−0.12	0.13	−0.6 ***	0.18
Age	−0.94 ***	0.31	−1.18 ***	0.35
Male	1.56 ***	0.35	4.40 ***	0.18
Unmarried	0.06	0.11	0.12	0.11
Educational attainment				
Middle school	−0.07	0.11	−0.31 **	0.12
High school and above	−0.12	0.13	−0.31 **	0.14
Household income	9.35 × 10^−8^	1.95 × 10^−7^	1.56 × 10^−7^	9.88 × 10^−7^
Living in urban area	−0.13	0.08	−0.11	0.14
Drink frequently	0.56 ***	0.12	0.37 ***	0.12
Obs	3908		3908	
Prob > chi2	*p* < 0.01		*p* < 0.01	

** *p* < 0.05, *** *p* < 0.01; Huber-White corrected standard errors.

**Table 5:** Marginal effects of unemployment rate on smoking behavior of employed individuals.

Variables	Marginal Effect
Smoking (*n* = 3908)	0.136 ***
Number of Cigarettes Smoked Daily (*n* = 3908)	
Non-smoker	−0.108 ***
1–10 a day	0.033 ***
10–20 a day	0.033 ***
More than 20 a day	0.042 ***

*** *p* < 0.01; Huber-White corrected standard errors.

Table 6 shows effects of unemployment rate on smoking of drinkers and non-drinkers, respectively. The area unemployment rate was positively associated with smoking behavior among individuals who were either drinkers or non-drinkers. Compared to drinkers, non-drinkers were more likely to smoke. Table 7 presents the marginal effects. In the ordered logic model, one percent increase in the unemployment rate increased the likelihood of smoking by 20.6 percent among non-drinkers and 4.6 percent among drinkers.

**Table 6:** Effects of unemployment rate on smoking behavior of drinkers and non-drinkers.

**Current Drinkers**				
**Variables**	**Smoking**		**Number of Cigarettes (Smoked Daily)**	
****	**Cof.**	**Std. Error**	**Cof.**	**Std. Error**
Unemployment rate	0.59 ***	0.20	0.52 *	0.27
Obs	1559		1559	
Prob > chi2	*p* < 0.01		*p* < 0.01	
**Non- Drinkers**				
**Variables**	**Smoking**		**Number of Cigarettes (Smoked Daily)**	
****	**Cof.**	**Std. Error**	**Cof.**	**Std. Error**
Unemployment rate	0.84 *	0.47	1.11 ***	0.41
Obs	3026		3026	
Prob > chi2	*p* < 0.01		*p* < 0.01	

* *p* < 0.1, *** *p* < 0.01; Huber-White corrected standard errors; The individual socio-economic status, health behavior and demographic variables were included.

**Table 7:** Marginal effects of unemployment rate on smoking behavior of drinkers and non-drinkers.

Variables	Non-Drinkers (*n* = 3026)	Current Drinkers (*n* = 1559)
Smoking	0.206 ***	0.046 ***
Number of Cigarettes Smoked Daily		
Non-smoker	−0.243 ***	−0.048 ***
1–10 a day	0.049 **	0.017 ***
10–20 a day	0.081 ***	0.014 *
More than 20 a day	0.113 **	0.017 *

* *p* < 0.1, ** *p* < 0.05, *** *p* < 0.01; Huber-White corrected standard errors.

Table 8 shows effects of unemployment rate on smoking by 2SLS. Since Hausman test statistics were not significant, we cannot reject the hypothesis that unemployment rate exogenous when dependent variable was smoking status. However, Hausman test indicated that unemployment rate was endogenous in the regression of number of cigarette smoked daily (Hausman test statistics are available at request). When estimated with 2SLS, the effects of unemployment rate on smoking were consistent. However, these effects were weaker after correcting for endogenous.

**Table 8:** Effects of unemployment rate on smoking behavior by 2SLS.

Variables	Smoking		Number of Cigarettes Smoked Daily	
	Cof. Std. Error		Cof. Std. Error	
Unemployment rate	0.03	0.07	0.32 *	0.17
Tobacco price	−0.04 *	0.02	−0.2 ***	0.05
Age	−0.16 ***	0.04	−0.45 ***	0.1
Male	0.28 ***	0.01	1.43 ***	0.03
Unmarried	0.02	0.02	0.06	0.04
Educational attainment				
Middle school	−0.006	0.02	−0.10 **	0.04
High school and above	−0.02	0.02	−0.13 **	0.05
Household income	2.33 × 10^−8^	3.81 × 10^−7^	9.37 × 10^−8^	8.54 × 10^−8^
Living in urban area	−0.02	0.01	−0.04	0.03
Drink frequently	0.12 ***	0.02	0.24 ***	0.04
Obs	4585		4585	
Prob > chi2	*p* < 0.01		*p* < 0.01	

* *p* < 0.1, ** *p* < 0.05, *** *p* < 0.01; Huber-White corrected standard errors.

## 5. Discussion

A strong positive association of the area-level unemployment rate with individual smoking was observed in China, even after controlling for demographic, social economic status, and alcohol consumption. In China, the increased unemployment rate provoked a shift from a preference towards the future to a preference leaning towards the present time, which resulted in increased smoking. Changes in smoking behavior resulting from financial burdens were less substantial. The growth of consumer incomes in China has been much faster than the increase in consumer expenditures on cigarettes in recent years. From 1990 to 2005, the former, on average, was about 2.8 times as much as the latter. The ability to pay for cigarettes has doubled as well [39].

According to most studies, smoking patterns appear to be procyclical in developed countries [6,11,12,13,14,15,16]. A decrease in smoking was followed when the unemployment rate increases. In contrast, a countercyclical trend was observed in China, which was consistent with those findings among individuals with lower social-economic status (e.g., construction workers and African Americans), old people or women in developed countries [7,8,9,10]. Combing our findings with existing literature, the positive association may be explained by the social supports that individuals enjoy [48]. Since the social safety net in China was not yet well developed, fluctuations in the unemployment rate tended to easily affect individual physical living conditions and increased the psychological stress [49]. It was possible that, without the protection from a sophisticated social welfare safety net, the high unemployment rate created anxiety for individuals, and as a result, trigged the increase in their smoking consumption. According to the economics theory, this was the case that individuals changed their time preference toward the present *versus* the future and coped the stress along with rising unemployment rate with smoking [24].

The effects of area-level unemployment rate on smoking behavior were stronger for those who were employed than the entire population, which is consistent with the literature that observes the negative influence of individual unemployment status on smoking behavior and that employed respondents were more likely to smoke than unemployed respondents with budget constraints [6,7].

Relative to their drinking counterparts, non-drinkers were more likely to smoke following the increased unemployment rate. Smoking and drinking were interdependent. When smokers also drank alcohol, they were likely to deal with the stress resulting from unemployment rate by increasing their alcohol consumption, whereas, given that drinking cannot be used to alleviate the pressure for smokers who do not drink, it is very plausible to expect that these smokers would increase their tobacco consumption more than their drinking counterparts [21,22].

Hausman test assisted that unemployment rate was not an endogenous variable when the dependent variable was smoking status, but the endogeneity problem existed between unemployment rate and number of cigarette smoked daily. Smoking behavior does not hurt labor market performance until the number of cigarette smoked daily reaches a certain limit [50]. In order to correct for the endogeneity of unemployment rate, we employed an instrumental variable (area-level unemployed insurance gold) and two-stage least squares (2SLS) estimation. A number of tests confirmed that the instrument was valid, and area-level unemployed insurance gold was negatively and significantly associated with unemployment rate. The results of the two-stage least squares (2SLS) estimation indicated the effects of unemployment rate on number of cigarette smoked daily were positive, but smaller than the results without correcting for endogeneity. A possible explanation for the difference between the endogeneity-corrected and uncorrected results was that individuals might get smoking earn penalty in labor markets [44,45].

Our finding highlights the need for multi-level approaches aimed at smoking reduction and cessation. Policy interventions for smoking-control should not only focus on the individual characteristics but also take the macro-economic environment into consideration. One intervention option is to implement different tobacco control policies based on the economic situations in areas or regions. The findings of positive impact of the area-level unemployment rate on smoking from our study indicate potentially effective policy strategies consisting of improving the social welfare safety net and increasing the cigarette tax in China, especially during economic recessions. The government should increase recourse allocation in health to strengthen the social welfare safety net to help ease economic hardship and mental stress. To achieve this, one option is to increase cigarette tax to make up for social welfare expenses and meantime adjust cigarette demand [51]. Other policies may include offering more smoking reduction and cessation services during economic recessions and strengthening anti-smoking education campaigns, especially during economic booms.

Our findings also have global public health implications. The vast majority of the world′s smokers live in low- and middle-income countries, which results in nearly 80 percent of deaths in middle- and low- income countries [1]. At present, global health is affected by economic fluctuation. Compared to developed countries, the social welfare safety net in developing countries is weaker [49]. Smoking behavior in developing countries is more easily affected by economic downturn. The gap between the numbers of smokers in developing countries *versus* developed countries is expected to widen [1]. As a result, special attention should be paid to smoking control in developing countries, especially during the economic recessions.

Our study has some limitations. The data sample only included those who were 45 years old or older; the findings may be generalized to the entire population with caution. Cigarette consumption may be higher among older populations who may not see the time preference of the future to the present as significant as younger people do. Young people may decrease their consumption for this reason. In addition, given their relatively low earnings, younger people may decrease their consumption simply because of the increase in cost of cigarettes. On the other hand, young people may not see the urgency of trading current smoking for the future healthy life because they are still healthy at a young age or their health stocks are high. There could be some wash-out effects between the two possibilities. In fact, we did run the analysis by dividing the sample into the working age group and retired group (the legal retirement age for women is 50 and the retirement age for men is 60) and did not find smoking behavior was significantly different between young people and old people. Further, the positive association of smoking and unemployment rate among retired people was comparable to that among working age people (in view of the article length, such results are not show in the article). Therefore, we, to some extent, believe that our findings are quite generalizable.

## 6. Conclusions

This is the first study to quantify the relationship between the area unemployment rate and individual smoking rate based on a large representative sample of China. Our findings indicate that the unemployment rate was positively related to smoking behavior in China. Economic hardship may worsen individual unhealthy behaviors including smoking. Smoking control and intervention strategies should take macroeconomic conditions such as variation of unemployment rate into consideration.
